# Physician Assurance Reduces Patient Symptoms in US Adults: an Experimental Study

**DOI:** 10.1007/s11606-018-4627-z

**Published:** 2018-08-20

**Authors:** Kari A. Leibowitz, Emerson J. Hardebeck, J. Parker Goyer, Alia J. Crum

**Affiliations:** Department of Psychology, Stanford University, Stanford, CA USA

**Keywords:** doctor-patient relationships, communication, allergy, patient centered care, psychosocial, placebo

## INTRODUCTION

Research on placebo effects demonstrates that physicians’ words powerfully influence patient health outcomes, catalyzing healing responses in conjunction with inactive treatments.^1^ But what are the effects of physicians’ words alone? In the quarter of doctor’s office visits in the USA in which medication goes unmentioned,^2^ what physicians say to patients may be especially impactful. However, research on the power of positive words from the provider is mixed. Some studies indicate that consultations including positive assurance lead to improved patient health,^3^ while others find no effect of physician assurance on patient outcomes.^4^ As physicians face increasing time demands, including higher patient volume and more electronic medical record documentation,^5^ the need to quantify the benefit of the many primary care visits in which medication is not discussed is paramount.^2^ The present study examined the effect of physician assurance absent pharmacological treatment—in this case, a simple sentence in which physicians told patients their symptoms would diminish—on patient allergic reactions.

## METHODS

**Fig. 1:**
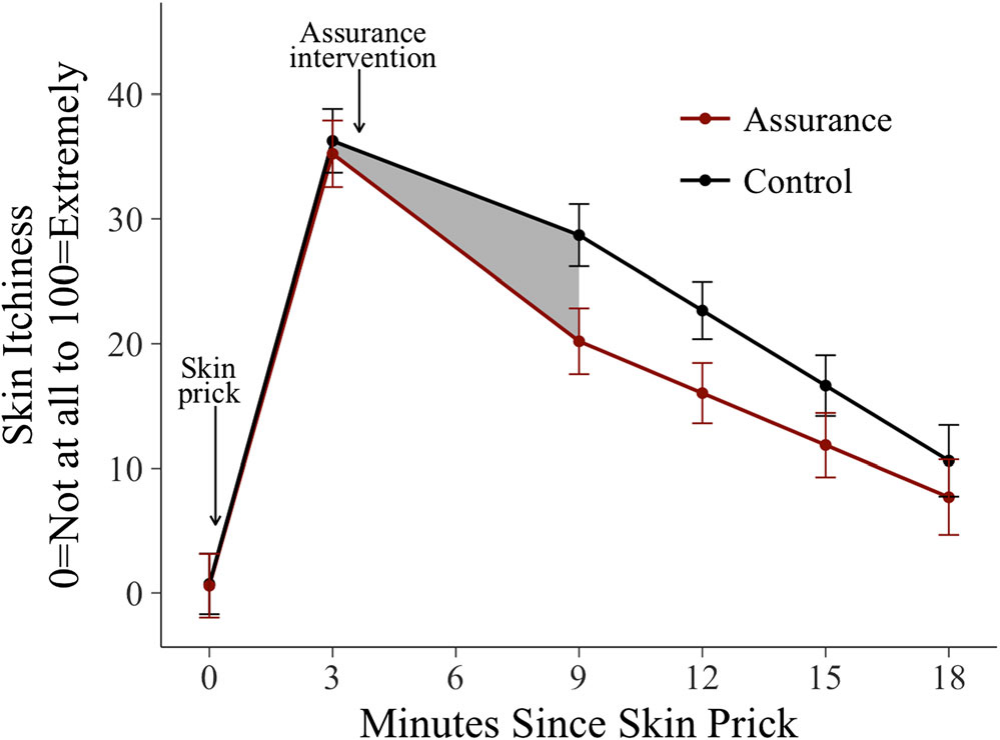
Longitudinal model predicting changes in patient skin itchiness ratings over time, as a function of condition. Lines represent estimated mean participant ratings of skin itchiness/irritation. Error bars reflect ± 1standard errors of the mean. Nonoverlapping error bars indicate reliable differences in itchiness level at the *p* < .05 level. The shaded area indicates a reliable difference in slopes (change in itchiness) at the *p* < .05 level. *N* = 76.

A healthcare provider administered a histamine skin prick^6^ using a Quintip lancet soaked in 10 mg/mL histamine dihydrochloride to the forearm of 76 participants (61.8% female; 54% under 22; 40.8% White, 23.6% Asian, 9.2% Hispanic/Latino, 9.2% African-American, 15.7% Other). Participants rated itchiness/irritation immediately before and at 3, 9, 12, 15, and 18 min post histamine skin prick. After the 3-min rating, the provider visually examined participants’ reactions. For participants (*N* = 36) randomized to “assurance” condition, the provider assured participants: “From this point forward your allergic reaction will start to diminish, and your rash and irritation will go away.” For participants (*N* = 41) randomized to control condition, the provider made no remarks about the reaction. Concealed allocation was used: the provider was blind to participant condition during application of histamine and learned participants’ condition, generated randomly by computer, immediately before the assurance intervention. The provider was not present during participant ratings. Multilevel longitudinal regression models used time-varying predictors to model condition differences in itchiness level and slope before and after physician assurance (Fig. 1). Two-tailed *z* scores were computed and *P* values ≤ .05 were considered statistically significant. Stanford IRB approved these procedures. Data and detailed protocol are available at https://osf.io/ryhsz/.

## RESULTS

During the 3 min after the histamine skin prick (but before physician assurance), itchiness increased similarly for both conditions (*B*_adj,*T*_ = 11.85 units/min vs. *B*_adj,*C*_ = 11.55 units/min; **Δ***B* = − 0.30, *z* = − 0.22, *p* = .83, 95% CI − 2.96 to 2.37, *d* = − 0.05). After the physician assurance, itchiness declined significantly faster in the assurance condition (*B*_adj,*T*_ = − 2.51 units/min vs. *B*_adj,*C*_ = − 1.26 units/min; **Δ***B* = − 1.24, *z* = − 1.96, *p* = .05, 95% CI − 2.49 to 0, *d* = 0.39) (Fig. 1). Consequently, participants who received assurance felt significantly less itchy immediately after the physician encounter at 9 min post-skin prick (0.48 standard deviations lower) than control participants (*M*_adj,*T*_ = 20.20 vs. *M*_adj,*C*_ = 28.70; *B* = − 8.50, *z* = − 2.34, *p* = .019, 95% CI − 15.62 to − 1.38, *d* = − 0.48). After minute 9, itchiness declined in both conditions by a similar amount (*B*_adj,*T*_ = − 2.01 units/min vs. *B*_adj,*C*_ = − 1.39 units/min vs; **Δ***B* = 0.62, *z* = −1.43, *p* = .154, 95% CI − 0.23 to 1.47, *d* = 0.31), such that the condition difference was also present at minute 12 (*M*_adj,*T*_ = 16.03 vs. *M*_adj,*C*_ = 22.67; *B* = −6.64, *z* = −1.99, *p* = .047, 95% CI − 13.19 to −0.08, *d* = − 0.37) but subsided by minute 15 (*M*_adj,*T*_ = 11.86 vs. *M*_adj,*C*_ = 16.64; *B* = −4.78, *z* = −1.35, *p* = 0.178, 95% CI − 11.73 to 2.17) (Fig. 1). That assurance had an immediate effect that weakened over time is unsurprising given the reaction was expected to decline over time in both conditions.^6^

## DISCUSSION

A brief, one-sentence assurance from a physician in response to an allergic reaction significantly reduced participants’ ratings of itchiness/irritation compared to a control condition that received no assurance (Fig. 1). Importantly, this effect was achieved without offering medication or other treatment. These results provide empirical support for the clinical utility of assurance alone and suggest that reassuring patients who consult for minor complaints may not only equip patients with helpful information—it may assist in alleviating patients’ symptoms. We suspect the present study is a conservative test of this effect since participants were healthy volunteers whose allergic reactions were unlikely to be highly stressful or concerning, and allergic reactions were expected to decline over time even without intervention. Physician assurance is a component of medical care that is surely familiar to physicians, yet is under-researched and often under-appreciated. Although meeting with patients for issues not ultimately requiring medication or treatment may be seen as costly or unnecessary from a health economics perspective, this study highlights the critical yet rarely quantified healing effect of visits in which the physician’s sole role is to assure patients they will soon feel better.
